# Developing a prognostic model of glutamine metabolism-related genes associated with clinical features and immune status in melanoma

**DOI:** 10.3389/fonc.2025.1485006

**Published:** 2025-08-20

**Authors:** Hongyan Hu, Jing Yang, Jin Miao, Chen Li, Cao Wang, Fengming Ran, Jie Zou, Yi Zhang, Liufang Zhao, Wentao Zhao, Conghui Ai

**Affiliations:** ^1^ Department of Pathology, Yunnan Cancer Hospital, The Third Affiliated Hospital of Kunming Medical University, Peking University Cancer Hospital Yunnan, Kunming, China; ^2^ Department of Oncology, First People’s Hospital of Kunming, Kunming, China; ^3^ Scientific Research Department, Yunnan Cancer Hospital, The Third Affiliated Hospital of Kunming Medical University, Peking University Cancer Hospital Yunnan, Kunming, China; ^4^ Department of Orthopedics, Yunnan Cancer Hospital, The Third Affiliated Hospital of Kunming Medical University, Peking University Cancer Hospital Yunnan, Kunming, China; ^5^ Department of Gynecology, Yunnan Cancer Hospital, The Third Affiliated Hospital of Kunming Medical University, Peking University Cancer Hospital Yunnan, Kunming, China; ^6^ Department of Head and Neck Cancer, Yunnan Cancer Hospital, The Third Affiliated Hospital of Kunming Medical University, Peking University Cancer Hospital Yunnan, Kunming, China; ^7^ Department of Gastrointestinal Oncology, Yunnan Cancer Hospital, The Third Affiliated Hospital of Kunming Medical University, Peking University Cancer Hospital Yunnan, Kunming, China; ^8^ Department of Radiology, Yunnan Cancer Hospital, The Third Affiliated Hospital of Kunming Medical University, Peking University Cancer Hospital Yunnan, Kunming, China

**Keywords:** glutamine metabolism, melanoma, prognosis, immune microenvironment, bioinformatics

## Abstract

**Introduction:**

Melanoma exhibited a poor prognosis due to its aggression and heterogeneity. The effect of glutamate metabolism promoting tumor progression on cutaneous melanoma remains unknown. Herein, glutamine metabolism-related genes (GRGs) were identified followed by constructing a prognostic model for melanoma via bioinformatics analysis.

**Methods:**

Patient data were collected from ,Gene Expression Omnibus (GEO) and The Cancer Genome Atlas—Skin Cutaneous Melanoma (TCGA-SKCM). In addition, GRGs were extracted from the MSigDB database, and the R package "Seurat" was used for scRNA-seq data processing.

**Results:**

eight key genes (CHMP4A, IFFO1, ANKRD10, ZDHHC11, CLPB, ANKMY1, TCAP and POLG2) were identified to construct a risk model. Based on univariate and multivariate Cox regression analyses, clinical characteristics including Clark stage and ulcer status were identified as independent prognostic factors, and a nomogram was successfully constructed. Survival analysis demonstrated that the overall survival rates of the high-risk group were lower than those of the low-risk group. The gene set enrichment analysis (GSEA) results showed that only ANKRD10, ANKMY1 and TCAP were enriched in the “glycolysis gluconeogenesis” pathway. The high-risk and low-risk groups displayed significant differences in immune cell infiltration and immune checkpoint expression. Analysis on drug sensitivity revealed that the high-risk group was highly sensitive to rapamycin. Additionally, it was verified that IFFO1, ANKRD10 and POLG2 were markedly upregulated and CHMP4A was also markedly downregulated in A375 cells by RT-PCR, which was consistent with the partial results of biological analysis.

**Discussion:**

Overall, it would provide valuable information about the GRGs of prognosis and immune status in melanoma.

## 1 Introduction

Melanoma has high malignancy and propensity for metastasis, triggering widespread interest (1). There is an increasing incidence of melanoma in teenagers and middle-aged people. Only 14% of cutaneous melanoma (CM) patients with metastasis reach survival beyond 5 years (2). Standard chemotherapy is largely ineffective against advanced or metastatic melanoma (3). In recent years, targeted immunotherapy has shown significant efficacy in advanced melanoma patients, but the 5-year survival rate of the patients still remains low (4), and tumor heterogeneity and drug resistance are the primary causes (5). Therefore, it is necessary to comprehensively study the mechanisms of tumorigenesis so as to explore new potential molecular biomarkers, which could be crucial for early diagnosis, targeted treatment, and prognosis assessment of melanoma patients.

Glutamine (Gln), as the most abundant non-essential amino acid, plays a pivotal role in energy metabolism. As a key source of carbon and nitrogen, it promotes tumor cell biosynthesis, energy production, and cellular homeostasis (6, 7). Gln metabolism, as an alternative source, can promote the tricarboxylic acid cycle in cancer cells and facilitate fatty acid synthesis through reductive carboxylation (8). The proliferation of cancer cells is addicted to Gln metabolism. Cancer cells cannot survive due to the absence of exogenous Gln (9). Thus, Gln metabolism can be a target for anticancer therapy (10, 11). The inhibition of Gln metabolism can enhance the antitumor effect of anti-PD-1 and the cytotoxic function of effector T cells (12, 13). One study has indicated a correlation between Gln and glycolysis in melanoma, highlighting the regulatory role of Gln metabolism in melanoma progression (14). Therefore, the genes related to Gln metabolism should be further investigated to predict treatment efficacy and clinical prognosis.

Thus, melanoma-related data from public databases were used to identify prognostic genes associated with Gln in melanoma patients via bioinformatics methods. A prognostic model was constructed to analyze the biological pathways associated with these prognostic genes. The relationships among clinical characteristics, the immune microenvironment, and drug sensitivity were established. This study focused on developing novel immunotherapy, targeted therapy strategies, and valuable prognosis of melanoma.

## 2 Materials and methods

### 2.1 Data sources

The Cancer Genome Atlas (TCGA) skin cutaneous melanoma (SKCM) dataset, which included 98 primary and 356 metastatic melanoma samples with survival information, was retrieved from UCSC Xena (https://xenabrowser.net/datapages/). The GSE46517 (GPL96) dataset, which contained 31 samples of primary melanoma, 73 samples of metastatic melanoma, and seven control samples, was mined from the Gene Expression Omnibus (GEO) database (https://www.ncbi.nlm.nih.gov/geo/). TCGA-SKCM and GSE46517 datasets were used as training sets 1 and 2, respectively. The validation set GSE65904 (GPL10558), which contained survival information of 210 melanoma tumor samples, and the single-cell dataset GSE72056 (GPL18573), which contained gene expression data for 4,645 quality-controlled (QC) cells, were obtained from the GEO database. Gln metabolism-related genes (GRGs) were mined from the Molecular Signatures Database (MSigDB) (https://www.gsea-msigdb).

### 2.2 Identification of differentially expressed genes and gene enrichment analysis

The differentially expressed genes (DEGs) between the melanoma and control groups in training set 2 were selected using the R language limma package (v 3.52.4) (15), with adj. *p* < 0.05 and |log_2_FC| ≥ 0.5. Moreover, the enrichment analysis of Gene Ontology (GO) [including cellular component (CC), molecular function (MF), and biological process (BP) analyses] and Kyoto Encyclopedia of Genes and Genomes (KEGG) enrichment analyses of the DEGs were completed using the clusterProfiler package (v 4.4.4) (16). The single-sample gene set enrichment analysis (ssGSEA) algorithm of the GSVA package (v 1.44.5) (17) was applied to compute the Gln metabolism score (GMS) in all samples of training set 1, with GRGs serving as the background gene set. Then, all genes of training set 1 were assigned to modules utilizing weighted gene coexpression network analysis (WGCNA) (v 1.71) (18). Modules relevant to GMS (*p* < 0.05) were confirmed as key modules, and key module genes were utilized for subsequent analyses.

### 2.3 Single-cell sequencing data analysis

Data from the single-cell dataset were integrated using the Seurat package for R (v 4.3.0) (19), with QC for the number of genes contained in the cells >1,700, housekeeping expression (corrected) >3, and all genes expressed >2% in at least five cells. The vst method was selected to screen the top 2,000 highly variable genes for downstream analysis. Subsequently, Uniform Manifold Approximation and Projection (UMAP) was utilized to reduce the dimensions. Using GRGs as the background gene set, the RcentageFeatureSet was utilized to calculate the percentage of GRG expression levels in each cell, and all cells were categorized into high- and low-expression groups to select intercellular differentially expressed GRGs (DE-GRGs) according to the median percentages.

### 2.4 Screening and analysis of candidate genes

Overlapping DEGs and key module genes were obtained from intersecting genes. Based on the intersecting genes, a protein–protein interaction (PPI) network was constructed. Then, the correlations between the intersecting genes and the DE-GRGs were computed according to Pearson’s correlation in training set 2, and the genes with |r| > 0.3, at least one differential GRG, and *p* < 0.05 were retained as candidate genes.

### 2.5 Construction and validation of the risk model

Based on the candidate genes via the glmnet package (v 4.1-4), key genes were selected via univariate Cox regression and least absolute shrinkage and selection operator (LASSO) regression analyses. Then, according to the correlation between expression of key genes and overall survival (OS), a risk model was constructed via LASSO (19). Risk scores were assessed utilizing the following formula:


$$risk\;score=\sum_{n=1}^{n}\left(coefi*Xi\right)$$

where coef and X indicate coefficients and gene expression, respectively. Moreover, the samples of training set 1 and the validation set were sorted into the high- and low-risk groups based on the median risk score. The Kaplan–Meier (K–M) survival curves were drawn using the survival package (v 3.4-0) for both risk groups in training set 1 and the validation set (20). To further assure the validity of the risk model, receiver operating characteristic (ROC) curves were generated at 3, 5, and 7 years, and the area under the curve (AUC) values were computed using survivalROC (v 0.4) (21).

### 2.6 Independent prognostic analysis and correlation analysis of clinical characteristics

Risk scores and seven clinical characteristics (age, sex, Clark stage, metastasis status, Breslow status, and ulcer status) were entered into the risk model for univariate and multivariate Cox regression analyses. Then, independent prognostic factors were selected to construct a nomogram via rms (v 6.3-0) (22). The 3-, 5-, and 7-year survival rates were predicted depending on the total points (the higher the points, the lower the survival rate). The predictive ability of the nomogram was assessed using calibration curves. Correlations between the risk score and eight clinical characteristics were analyzed via correlation analysis.

### 2.7 GSEA

To understand prognostic gene-related biological functions and signaling pathways, the correlations between prognostic genes and other genes were calculated and sequenced separately in training set 1. Based on the C2:KEGG gene set downloaded from the msigdbr package in R (v 7.5.1), the sequenced genes were enriched using the GSEA function in R (adj. *p* < 0.05).

### 2.8 Immune microenvironment analysis and regulatory networks for prognostic genes

The immune-related genes identified in the literature were used as background gene sets (23), and the samples in the training set were analyzed using ssGSEA to obtain enrichment scores for 28 immune cell types. Differences in enrichment scores for each immune cell between the melanoma and control groups were analyzed via the Wilcoxon test. The stromal score, immune score, and ESTIMATE score (summed over the first two) of the samples in training set 1 were estimated using the estimate package in R (v 1.0.13). Moreover, the expression of common immune checkpoints, including PD-L1, CTLA-4, LAG-3, GAL9, TIM-3, PD-1, PD-1LG2, and TIGIT, was compared between the high- and low-risk groups. Prognostic gene-related miRNAs were predicted using the starBase database.

### 2.9 Drug sensitivity analysis

The 50% inhibitory concentration (IC_50_) values of 198 chemotherapeutic drugs were computed and compared according to the Genomics of Drug Sensitivity in Cancer database using the OncoPredict package (v 0.2).

### 2.10 Cell line culture

The cell lines A375 (primary cutaneous melanoma) and A2058 (metastatic melanoma), purchased from ATCC (cat. nos. CRL-1619 and CRL-11147), were cultured in Dulbecco’s modified Eagle’s medium (DMEM; #12634-010, USA) supplemented with 10% fetal bovine serum (FBS; No. SH30070.02, HyClone, Utah, USA) in incubators with 5% CO_2_ at 37°C. The human immortalized keratinocyte HaCaT cell line (CVCL-0038) as the control cell line was purchased from the Kunming Institute of Zoology and cultured in Dulbecco’s modified Eagle’s medium.

### 2.11 RT-qPCR

Total RNA was extracted from melanoma cell lines by TRIzol (15596018, Thermo, Beijing, China). A PrimeScript™ RT kit (R232-01, Vazyme, Nanjing, China) was applied to synthesize cDNA. Real-time polymerase chain reaction (RT-PCR) was achieved using SYBR Green Master Mix (Q111-02, Vazyme), and the expression levels were confirmed via the 2^−ΔΔCt^ method. The expression of each mRNA was standardized to the expression of GAPDH mRNA. All primers, as shown in **Supplementary Table 1**, were purchased from Tsingke Biotech (Beijing, China).

## 3 Results

### 3.1 Identification and functional analysis of DEGs

**Figure 1:**
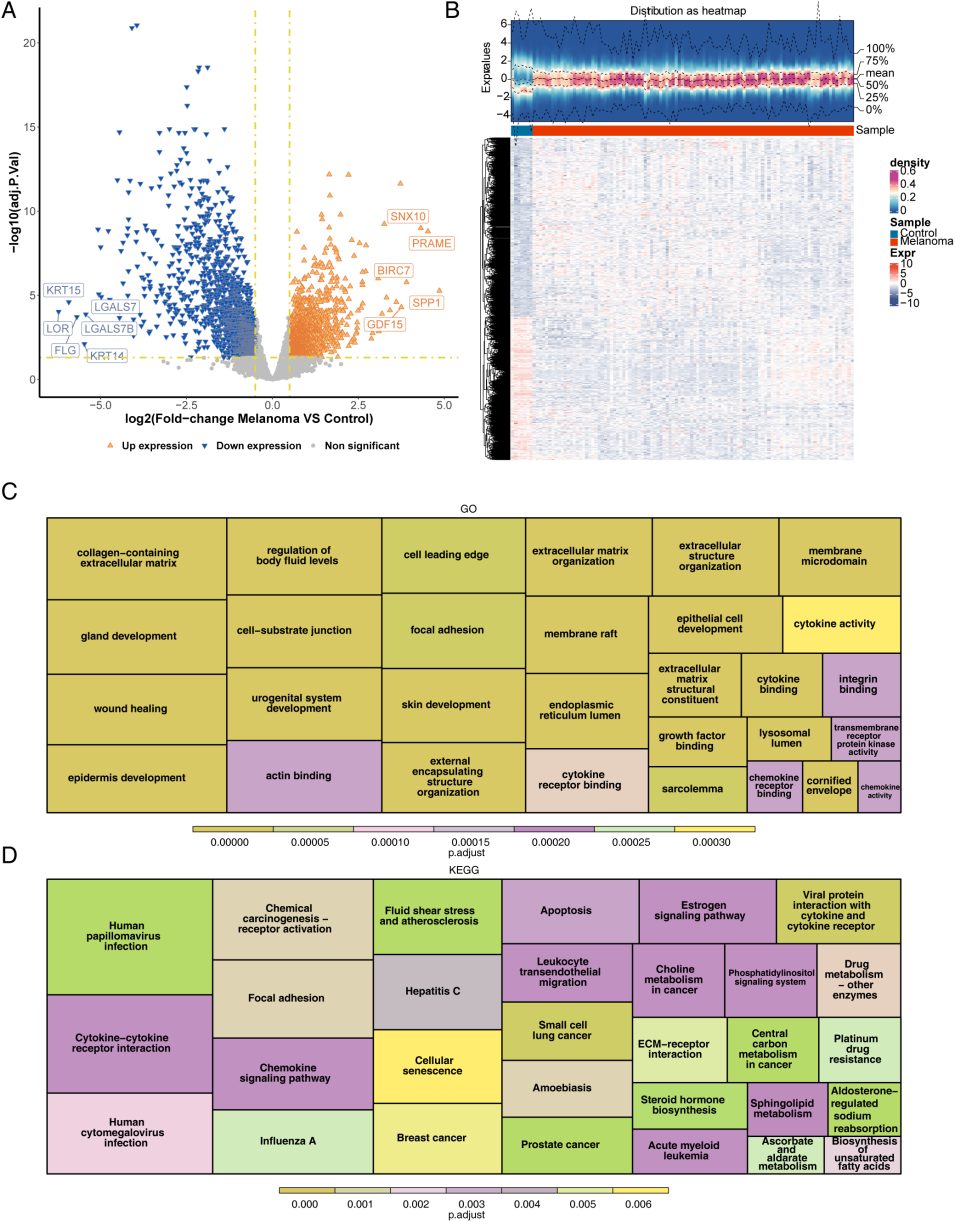
Identification and functional enrichment analysis of DEGs between melanoma patients and controls. **(A)** Volcano plot of DEGs between melanoma and control in GEO. *p* < 0.05 and |log_2_FC| ≥ 0.5 were used to identify significant DEGs. The red dots represent upregulated genes, and the blue dots represent downregulated genes. **(B)** Heatmap of DEGs. **(C)** Functional enrichment of differential genes for GO (displaying the top 10 functional items in each section). **(D)** Functional enrichment of differential genes for KEGG (displaying the top 30 functional pathways). DEGs, differentially expressed genes; GEO, Gene Expression Omnibus; log2FC, log2 fold change; GO, Gene Ontology; KEGG, Kyoto Encyclopedia of Genes and Genomes.

A total of 3,216 DEGs (1,784 upregulated and 1,432 downregulated) between the melanoma and control groups were selected from training set 2 (**Figures 1A, B**). Functional enrichment analysis indicated that DEGs were related to 1,238 GO terms (**Figure 1C**), including 83 CCs (e.g., “collagen-containing”, “extracellular matrix”, and “cornified envelope membrane raft”), 56 MFs (e.g., “cytokine binding”, “cytokine activity”, and “actin binding”), and 1,099 BPs (e.g., “epidermis development”, “skin development”, and “epithelial cell proliferation”). In addition, 76 functional pathways were enriched according to the KEGG analysis. The KEGG enrichment analysis results displayed that chemical carcinogenesis-receptor activation, focal adhesion, and apoptosis were the pathways enriched in the DEGs (**Figure 1D**).

### 3.2 WGCNA and the acquisition of intersecting genes

**Figure 2:**
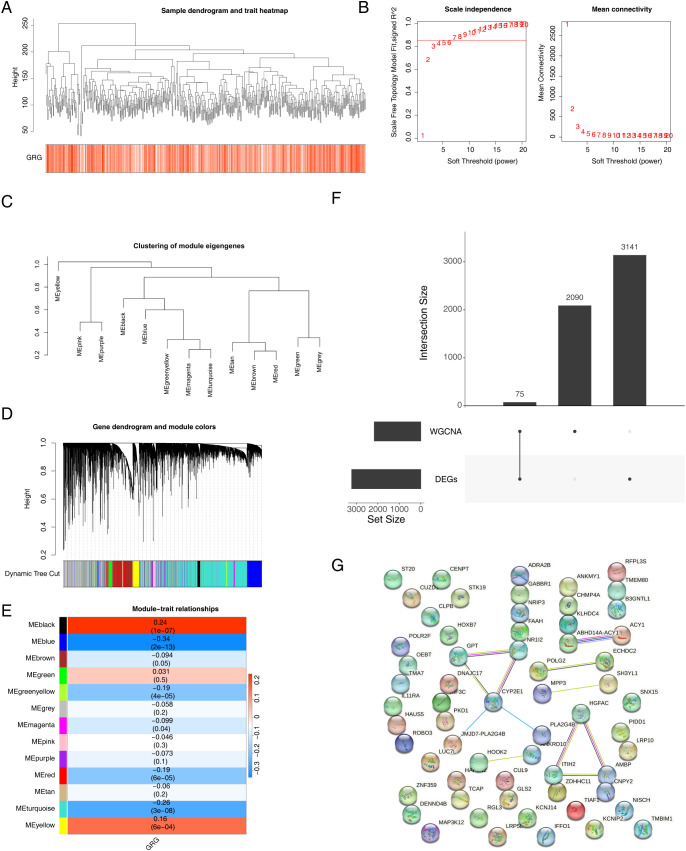
Melanoma-related genes were screened via WGCNA. **(A)** Sample clustering tree. **(B)** Analysis of the scale-free index for various soft-threshold powers (β). **(C)** The minimum number of genes per module was 300, and 13 modules were obtained when MEDissThres was equal to 0.2. **(D)** Cluster dendrogram of the coexpression network modules (1 − Topological Overlap Matrix (TOM)). **(E)** Analysis of correlations between the modules and melanoma; *p*-values are shown. **(F)** Wayne diagram of intersecting genes. **(G)** PPI network of significantly differentially expressed glutamine metabolism-related genes. WGCNA, weighted gene coexpression network analysis; PPI, protein–protein interaction.

First, samples from the training set were used to construct a clustering tree (**Figure 2A**). A soft threshold of 7 (R^2^ = 0.98) was applied to construct a scale-free network (**Figure 2B**). Then, an adjacency matrix and topological overlap matrix were constructed (**Figure 2C**). Finally, 13 modules were obtained based on average hierarchical clustering and dynamic tree clipping (**Figure 2D**). The blue module (|cor| = −0.34, *p* < 0.05, containing 2,165 module genes) was associated with GMS, which was identified as a key module (**Figure 2E**). Subsequently, 75 intersecting genes were obtained by overlapping DEGs and module genes (**Figure 2F**). A PPI network of 75 intersecting genes showed multiple pairs of relationships for intersecting genes. For example, *CYP2E1* was associated with multiple genes, such as *GPT* and *NR1I2* (**Figure 2G**).

### 3.3 Single-cell analysis and screening of candidate genes

**Figure 3:**
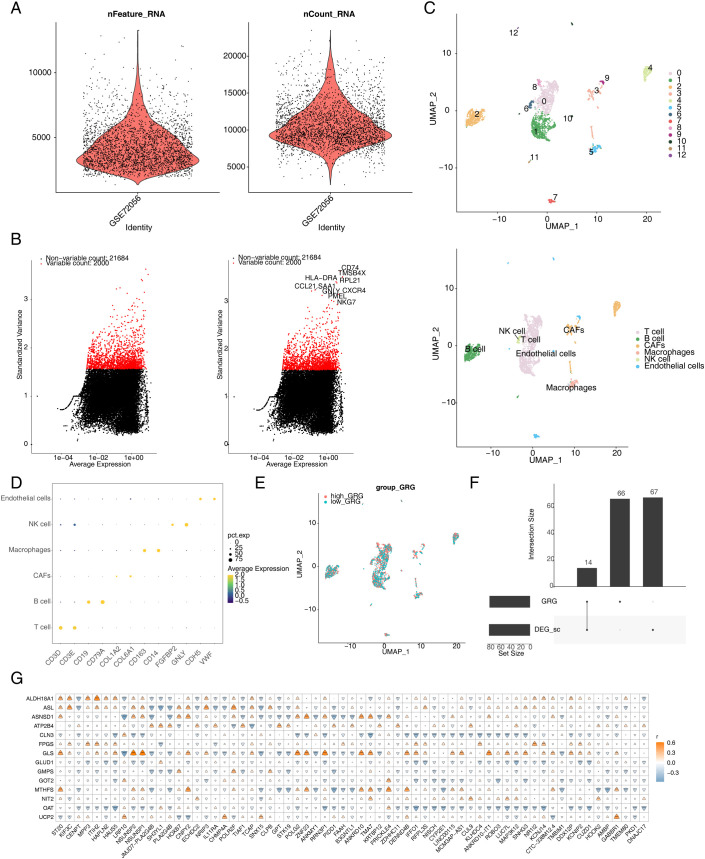
Annotation of cell subsets from single-cell sequencing data and identification of differentially expressed genes. **(A)** After quality control of scRNA-seq, 2,887 core cells and 23,684 genes were identified. **(B)** The variance diagram shows the variation of gene expression in all melanoma cells. The red dots represent highly variable genes, and the black dots represent non-variable genes. **(C)** shows the clustering and subgroup annotation results of single-cell analysis cells. **(D)** The bubble diagram shows the expression of marker genes for each cell cluster. **(E)** Glutamine metabolism score in each cell cluster. **(F)** Wayne diagram of glutamine metabolism-related genes. **(G)** Heatmap of candidate genes and differentially expressed genes. scRNA-seq, single-cell RNA-seq.

After QC, 2,887 cells and 23,684 genes remained in the single-cell dataset (**Figure 3A**). Then, 2,000 highly variable genes were selected for subsequent analysis (**Figure 3B**). The cells were clustered into 13 clusters based on distance and were annotated to six cell types [T cells, B cells, Cancer-Associated Fibroblasts (CAFs), macrophages, natural killer (NK) cells, and endothelial cells] via marker genes (**Figures 3C, D**). Furthermore, 81 DEGs were screened between the high- and low-expression groups (min.pct = 0.25, logfc.threshold = 0.25) and were crossed with 80 GRGs to obtain 14 DE-GRGs (*ALDH18A1*, *ASL*, *ASNSD1*, *ATP2B4*, *ALDH18A1*, *ASL*, *ASNSD1*, *ATP2B4*, *CLN3*, *FPGS*, *GLS*, *GLUD1*, *GMPS*, *GOT2*, *MTHFS*, *NIT2*, *OAT*, and *UCP2*) (**Figures 3E, F**). In addition, 65 candidate genes were obtained based on Pearson’s correlation analysis of the intersecting genes and DE-GRGs (**Figure 3G**).

### 3.4 Construction, evaluation, and validation of a risk model

**Figure 4:**
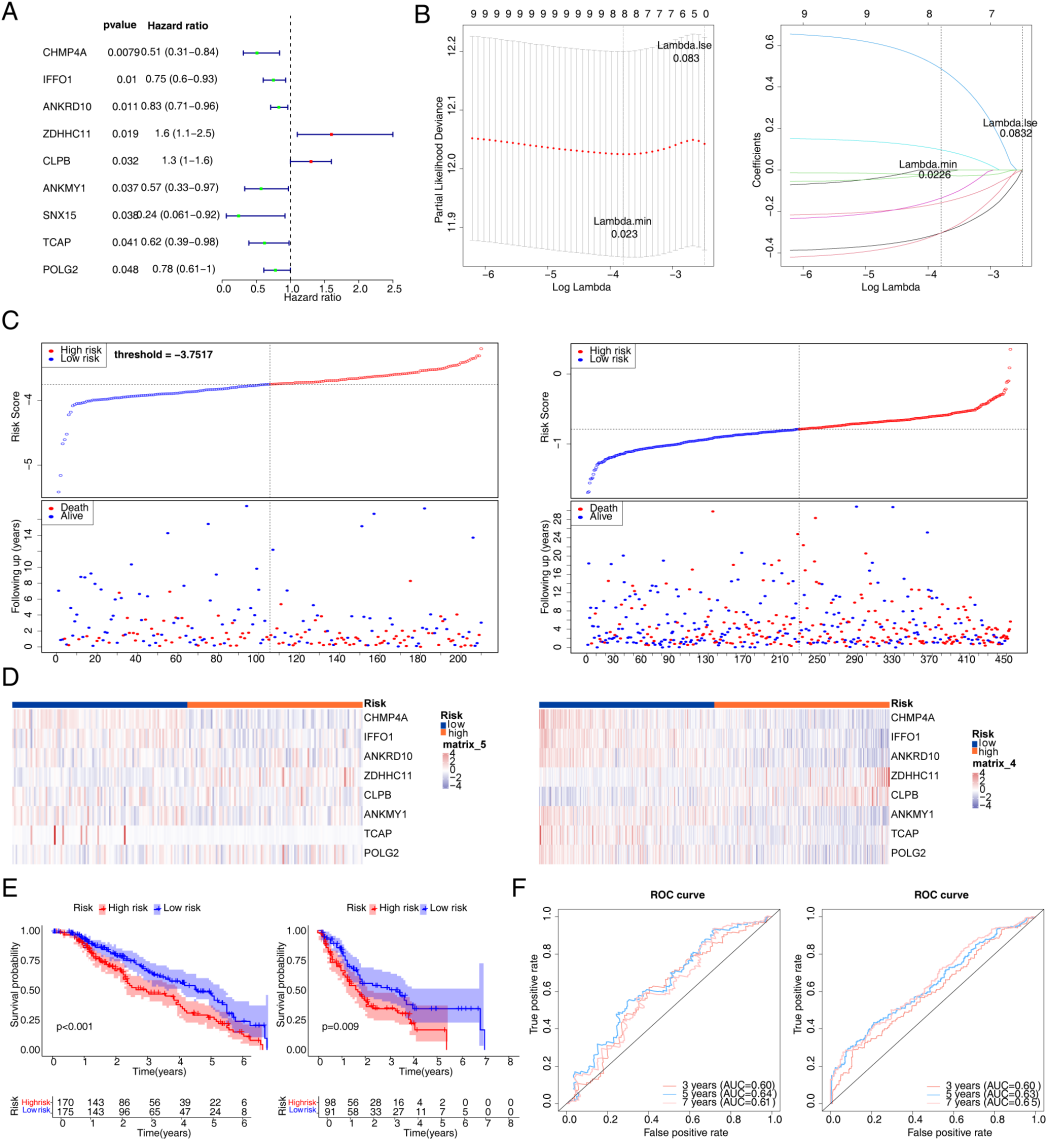
Construction of risk signature in TCGA cohort. **(A)** Univariate Cox regression analysis of OS. **(B)** LASSO regression of OS-related genes. **(C)** Risk survival status plot (C1 for training set 1; C2 for validation set). **(D)** The representative gene variants in the high- and low-risk groups (D1 for training set 1; D2 for validation set). **(E)** Kaplan–Meier curve (E1 for training set 1; E2 for validation set). **(F)** The AUC of the prediction of 3-, 5-, and 7-year survival rates of melanoma patients. TCGA, The Cancer Genome Atlas; OS, overall survival; LASSO, least absolute shrinkage and selection operator; AUC, area under the curve.

A total of nine genes were identified via univariate Cox regression analysis (**Figure 4A**), and further eight key genes (*CHMP4A*, *IFFO1*, *ANKRD10*, *ZDHHC11*, *CLPB*, *ANKMY1*, *TCAP*, and *POLG2*) were identified via LASSO based on 65 candidate genes (**Figure 4B**). Subsequently, a risk model was constructed according to the expression of eight key genes, and risk scores were also computed. Risk curves (**Figure 4C**) and gene expression data of the two risk groups were plotted based on risk scores (**Figure 4D**). It was observed from the K–M curves that the difference in the survival of melanoma patients was highly significant (*p* < 0.005) (**Figure 4E**). The AUC values exceeded 0.6 at 3, 5, and 7 years for melanoma patients. It suggested that the eight key genes could reliably predict survival status (**Figure 4F**).

### 3.5 Construction of an independent prognostic model and correlation analysis of risk scores and clinical characteristics

**Figure 5:**
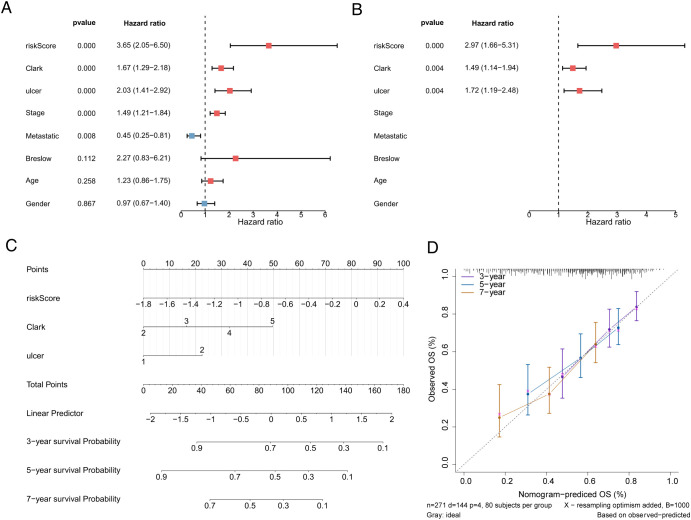
Nomogram to assess the risk of melanoma patients. **(A)** Univariate Cox analysis of risk scores and clinical characteristics. **(B)** Multifactorial Cox analysis. **(C)** Construction of the nomogram model. **(D)** The calibration curve of the nomogram.

**Figure 6:**
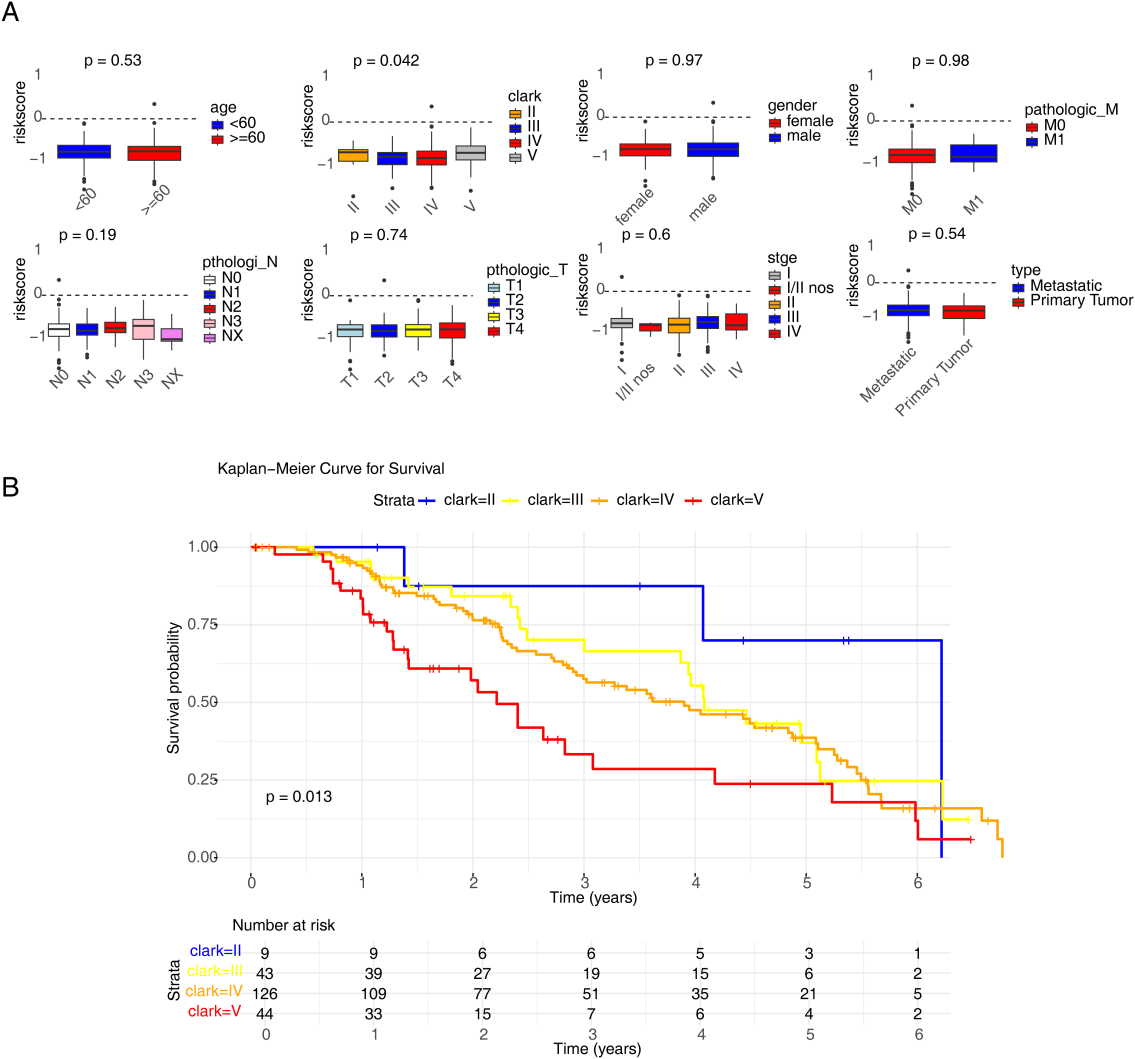
Correlation analysis of risk scores with clinical characteristics. **(A)** The analysis model of age, gender, Clark, stage, primary and metastatic tumors, Breslow, ulcer status, and the expression of CD274. **(B)** Kaplan–Meier curve result of Clark groups.

To screen independent prognostic factors, clinical characteristics and risk scores were subjected to univariate and multivariate Cox analyses. The risk score, Clark stage, and ulcer status were identified as independent prognostic factors, which were used to construct a nomogram (**Figures 5A–C**). The slope of each calibration curve was close to 1, indicating favorable prediction accuracy of the nomogram (**Figure 5D**). In addition, correlation analysis of seven clinical characteristics and CD274 expression demonstrated that major differences existed in Clark stage and CD274 expression in melanomas (*p* = 0.019 and *p <* 0.001, respectively; **Figure 6A**). There was a marked difference in the survival status of the samples in the Clark subgroups (**Figure 6B**).

### 3.6 GSEA in training set 1 and the landscape of the immune microenvironment in the two risk groups

**Figure 7:**
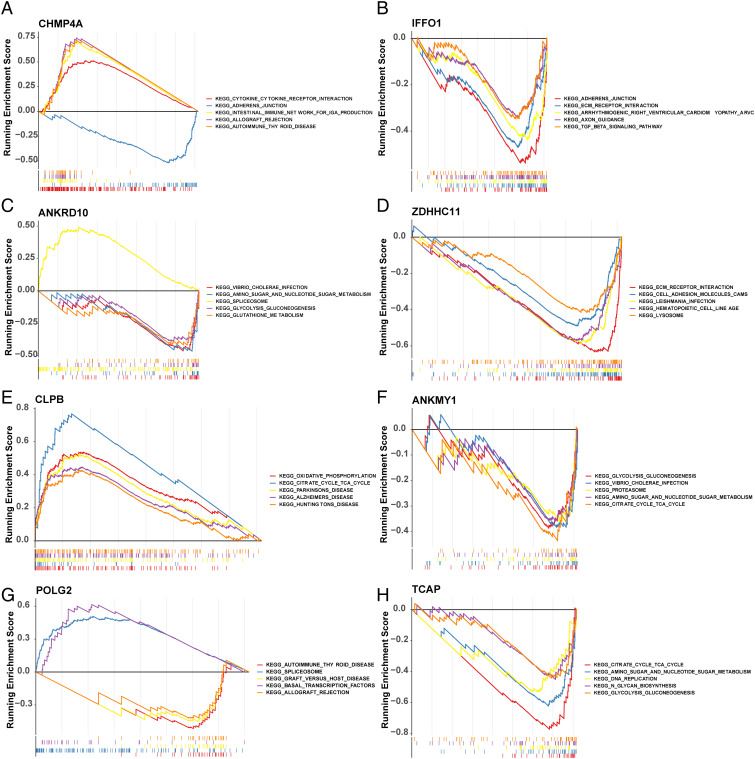
Biological characteristics between high- and low-risk groups. GSEA of GO and KEGG between high- and low-risk groups. GSEA, gene set enrichment analysis; GO, Gene Ontology; KEGG, Kyoto Encyclopedia of Genes and Genomes. **(A–H)** represent the GSEA results of CHMP4A, IFFO1, ANKRD10, ZDHHC11, CLPB, ANKMY1, POLG2, TCAP, respectively.

GSEA demonstrated that eight key genes were enriched in KEGG pathways (**Figure 7**).

**Figure 8:**
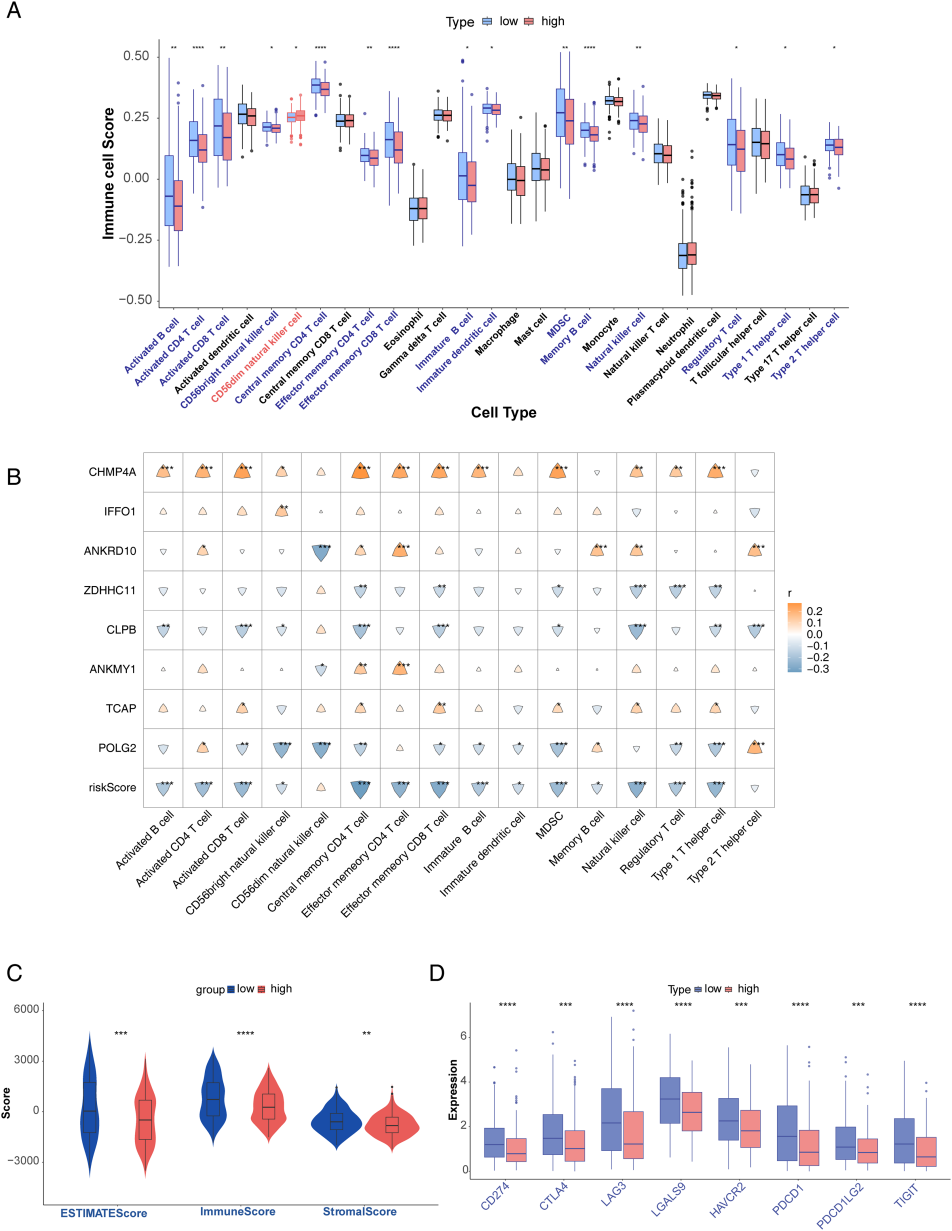
Analysis of immune microenvironment. **(A)** Differences in immune cell enrichment scores. **(B)** Correlation analysis of immune cells and prognostic genes. **(C)** ESTIMATE score and risk score, immune score and risk score, and stromal score and risk score. **(D)** Differential expression of immune checkpoints in high- and low-risk groups. *p<0.05, **p<0.01, ***p<0.001, and ****p<0.0001.

There were significantly different enrichment scores of the 16 immune cells between the high- and low-risk groups (*p* < 0.05). The expression of immune cells in the high-risk group, except for CD56dim NK cells, was higher than that of the low-risk group (**Figure 8A**). A heatmap of the correlation between the eight key genes and risk scores of the 16 immune cells was also drawn in **Figure 8B**. The stromal, immune, and ESTIMATE scores were substantially different between the two risk groups (*p* < 0.05), and all were low in the high-risk group (**Figure 8C**). In addition, all immune checkpoint genes exhibited low expression in the high-risk group (*p* < 0.001) (**Figure 8D**).

### 3.7 mRNA–miRNA network construction and drug sensitivity analysis

**Figure 9:**
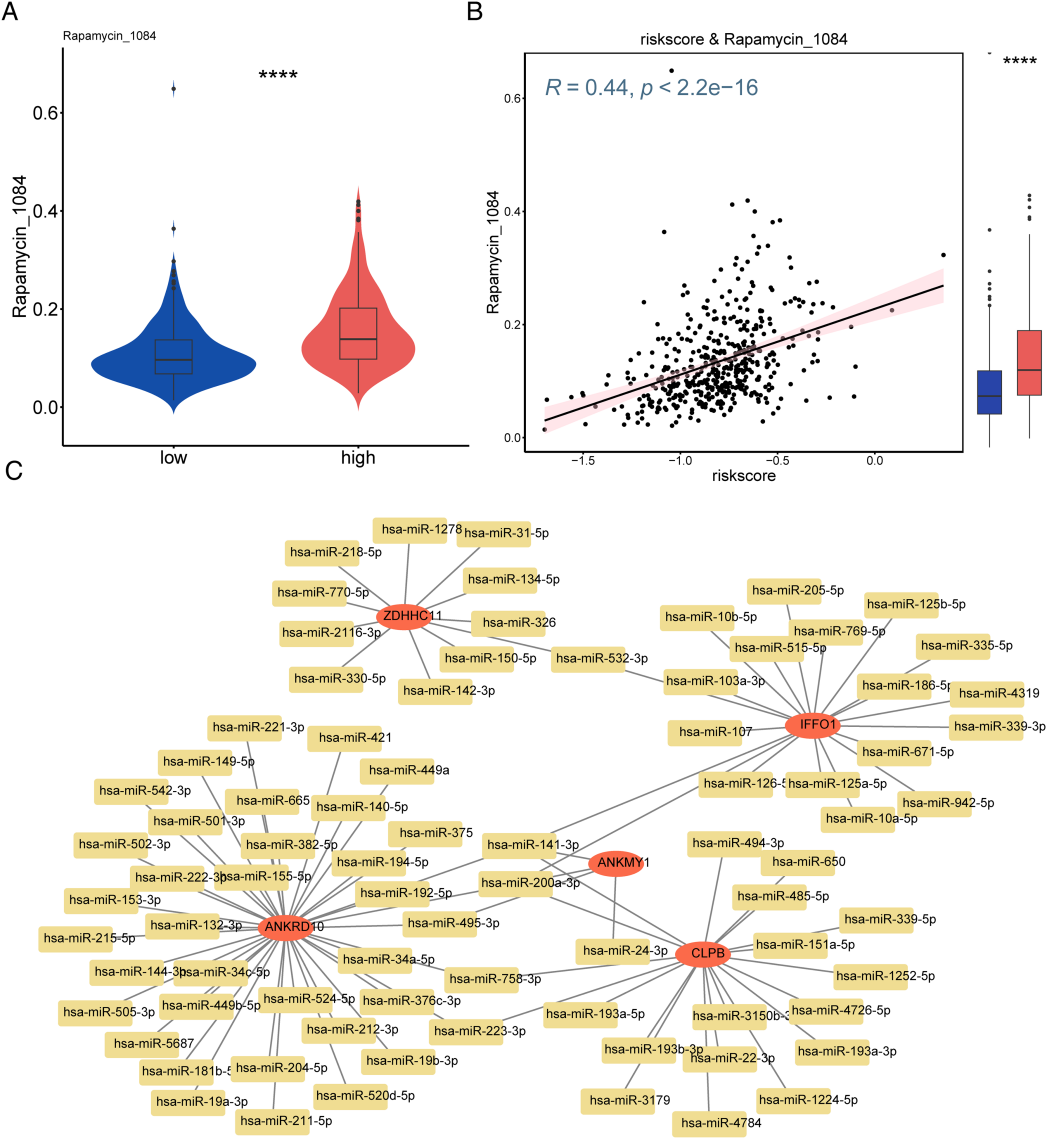
Screening of therapeutic agents for melanoma and constructed mRNA–miRNA network based on risk models. **(A)** Differences in drugs between high- and low-risk groups. **(B)** Scatter plot visualizing the correlation between drugs and risk scores. **(C)** Construction of the mRNA–miRNA interaction network. ****p<0.0001.

Drug sensitivity analysis revealed that the high-risk group was strongly sensitive to rapamycin (*p* < 0.0001, **Figures 9A, B**). A total of 81 miRNAs (e.g., hsa-miR-421, hsa-miR-449a, and hsa-miR-375) associated with the key genes were predicted using the starBase database (**Figure 9C**).

### 3.8 The expression of eight key genes in control individuals and patients with melanoma

**Figure 10:**
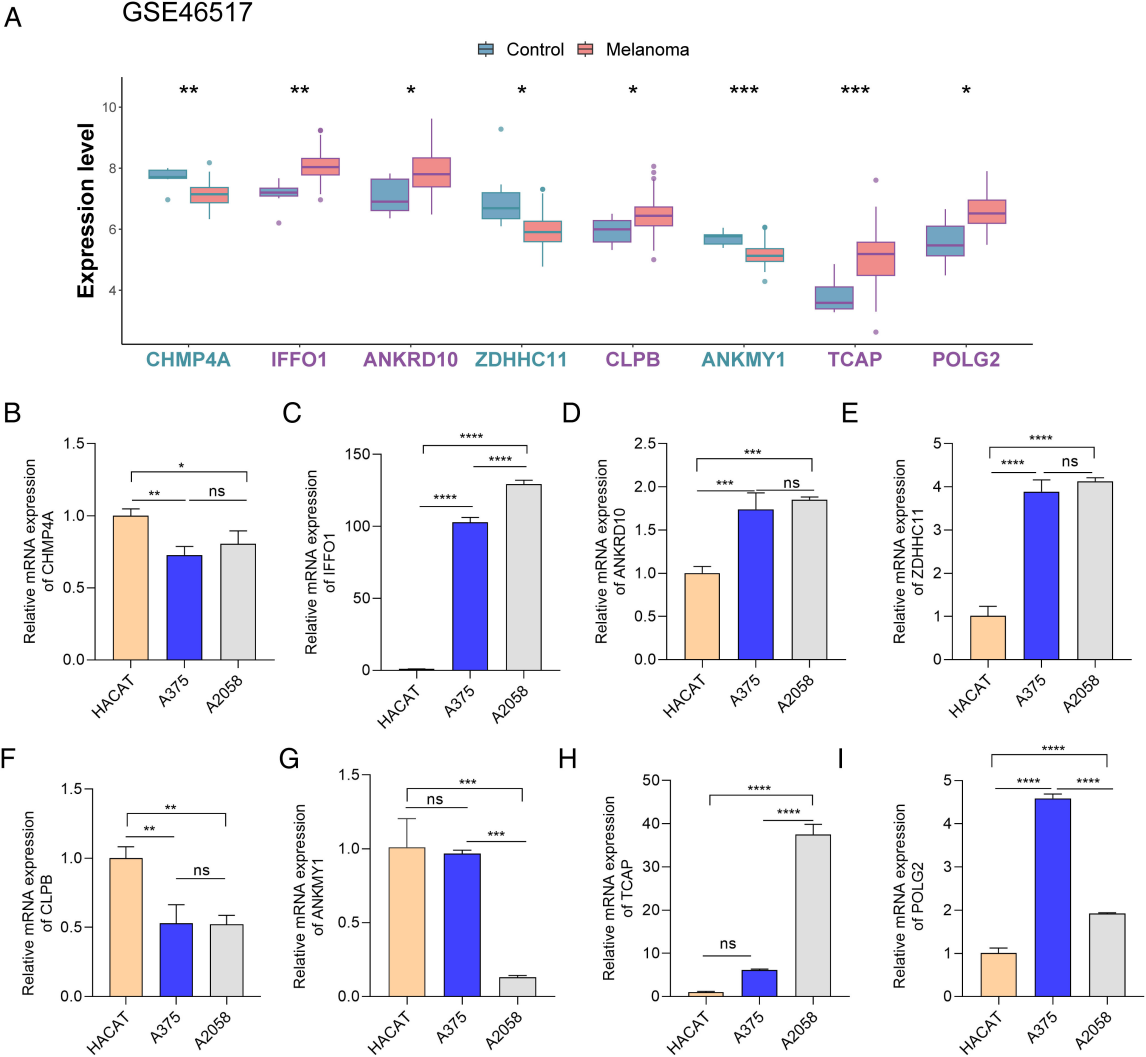
The expression of eight key genes in controls and melanoma patients. **(A)** The expression of eight key genes in GSE46517. **(B–L)** The expression of eight key genes between normal and melanoma cells (in HACAT, A375, and A2058cells). *p < 0.05, **p < 0.01, ***p < 0.001, and ****p < 0.0001. ns, *p* > 0.05.

In GSE46517, *IFFO1*, *ANKRD10*, *CLPB*, *TCAP*, and *POLG2* displayed high expression, but the expression levels of *CHMP4A*, *ZDHHC11*, and *ANKMY1* were low in the melanoma group (**Figure 10A**). The expression of these genes was further examined via RT-PCR in HACAT and melanoma cells (A375 and A2058). It was interesting that *IFFO1*, *ANKRD10*, and *POLG2* were markedly upregulated and that *CHMP4A* was also markedly downregulated in A375 cells (**Figure 10B**), which was partially consistent with the results of biological analysis.

## 4 Discussion

CM remains the most lethal form of skin cancer, with an annual increase of more than 3% (24). Gln metabolism plays a crucial role in tumor survival and progression (25). To elucidate key players in this pathway in melanoma, we integrated bulk RNA-seq and single-cell RNA-seq (scRNA-seq) data, identifying eight glutamine metabolism-related genes. Based on these genes, we developed a prognostic risk model that demonstrates robust performance in predicting glutamine metabolism activity and patient outcomes.

In this study, the eight-gene (*CHMP4A*, *IFFO1*, *ANKRD10*, *ZDHHC11*, *CLPB*, *ANKMY1*, *TCAP*, and *POLG2*) prognostic model had promising prognostic value, which was demonstrated by the ROC curve results. In addition, a nomogram combining prognostic models and clinicopathological factors accurately predicted the survival rate of melanoma patients at 3, 5, and 7 years. Based on the analysis of the relationship between the model and clinicopathological characteristics, the risk score was significantly associated with the Clark stage of melanoma patients and CD274 expression. It indicated that the model had predictive value for OS. The risk model demonstrated significantly reduced survival rates in high-risk patients, indicating a need for intensified therapeutic approaches. From an immunotherapy perspective, this model could contribute to identifying high-risk patients with poor immunotherapy response, prioritizing this subgroup for combined treatment in clinical practice. Crucially, prospective validation of the utility of the model in guiding treatment selection and prognosis management requires evaluation in clinical cohorts.

It was worth noting that the relationship between the eight key genes and glutamine metabolism in melanomas has not been reported. The abnormal expression of these genes in melanomas was further validated via RT-PCR. Among these genes, four genes (*IFFO1*, *ANKRD10*, *POLG2*, and *TCAP*) were significantly overexpressed, while two genes (*CHMP4A* and *ANKMY1*) had low expression via *in vitro* validation. *ZDHHC11* and *CLPB* exhibited inconsistent expression trends between TCGA data and melanoma cells. This discrepancy may be attributed to the use of melanoma cancer cell lines for PCR validation, while the dataset covered melanoma tissue (26). *IFFO1* is a non-homologous end-joining protein that plays a role in promoting the repair of DNA double-strand breaks (27). Previous studies have indicated that the expression levels of *IFFO1* were associated with tumor progression and immune infiltration (28). Recently, *IFFO1* inhibited tumor metastasis and reversed drug resistance through histone deacetylase and RNA methylation mechanisms in ovarian cancer (29). In our study, it was observed that *IFFO1* could have a promoting effect on melanoma cells. *ANKRD10*, as a protein-coding gene, has not been extensively studied. It was reported that *ANKRD10* affected antitumor activity by regulating morin treatment in tongue squamous carcinoma cells (30). *ANKRD10* acted as a DNA methylation-driven gene in glioblastoma (31). In our study, *ANKRD10* exhibited high expression in melanoma patients. *POLG2* was essential for mammalian embryogenesis and mtDNA maintenance (32). However, the underlying molecular basis and functional significance of *POLG2* in tumors were unknown. It may achieve unexpected results for the treatment and prognosis of tumors. Microarray analysis demonstrated that *CHMP4A* was used as a prognostic biomarker and druggable target for various diseases such as hepatocellular carcinoma, colorectal cancer, and ovarian carcinoma (33–35). *CHMP4A* revealed low expression in melanoma as a prognostic gene in our study. *ZDHHC11*, a member of the DHHC palmitoyl transferase family, regulated innate immune response to DNA virus by mediating the *IFN-β* promoter (36). In our study, *ZDHHC11* was an unfavorable factor in melanoma and related to immune cells. There are little data on *CLPB*, *TCAP*, and *ANKMY1* in malignancies. Defects in *CLPB* could cause neurological involvement and neutropenia (37). *TCAP* plays a role in cell adhesion and energy regulation of synaptogenesis in the vertebrate nervous system (38). *ANKMY1*, as a component regulating cytoskeleton organization, has not been reported in tumors (39). Combined with the above findings, it was first reported that the above GRGs could be closely related to the prognosis of melanoma.

ssGSEA displayed obvious differences in immune cell subpopulations between the high-risk and low-risk subgroups. It suggested that immune cells and immune function were related to GRGs in melanoma patients. The risk scores of eight prognostic genes varied among different immune cells in this work. Glutamine antagonism led to plasticity in the metabolism of cancer cells and effector T cells, which could become a new target for tumor immunotherapy (40). Some immune cells could promote antitumor immunity or have immunosuppressive effects in melanoma (41). Importantly, all immune checkpoint genes in our study exhibited low expression in the high-risk group. The reprogramming of glutamine metabolism regulated immune escape by modulating the expression of tumor PD-L1 in tumors (12). These results indicated that high-risk melanoma patients are intolerant to immunotherapy, resulting in poorer prognoses.

Rapamycin primarily inhibited melanoma by targeting the mTOR pathway (42). Through FKBP12-mediated suppression of mTORC1 activity, it reduced S6K and 4E-BP1 phosphorylation, thereby blocking tumor proliferation. Concurrently, rapamycin relieved mTORC1-mediated autophagy suppression and shifted cellular metabolism toward catabolic states (43). It further remodeled the tumor microenvironment via immune modulation and anti-angiogenesis while exhibiting synergy with pathways like MAPK (44, 45). Crucially, our drug sensitivity analysis revealed the remarkable effectiveness of rapamycin in high-risk melanoma subgroups. This differential response implied unique molecular dependencies, particularly mTOR network vulnerabilities in aggressive tumors. It displayed therapeutically exploitable selectivity beyond the canonical mechanisms of rapamycin. Collectively, these findings indicate that rapamycin exerts multi-targeted inhibitory effects on melanoma cell proliferation, survival, and the tumor microenvironment. This provides a theoretical foundation and identifies potential therapeutic targets for precision treatment In high-risk melanoma patients.

In summary, our study identified a significant correlation between the eight key genes and risk scores of immune cells/checkpoints in melanoma. Nevertheless, several important limitations are worth considering, such as requiring further validation in independent clinical cohorts and unresolved regulatory mechanisms of signature genes.

## 5 Conclusion

By integrating scRNA-seq and bulk RNA-seq data, multiple machine learning methods were applied to develop a novel prognostic model for predicting OS in melanoma patients. The model could be used to estimate the survival probability of melanoma patients. Additionally, the risk score of this model as an independent prognostic factor was strongly associated with Gln metabolism and clinicopathological characteristics. Overall, it could provide a reliable predictor of melanoma efficacy and potential avenues for the targeted treatment of melanoma in the future.

## Data Availability

The original contributions presented in the study are included in the article/**Supplementary Material**. Further inquiries can be directed to the corresponding authors.
