# Supplementary material for: Developing a prognostic model of glutamine metabolism-related genes associated with clinical features and immune status in melanoma
Source: Front Oncol. 2025 Aug 20;15:1485006. doi: 10.3389/fonc.2025.1485006 (PMC12405417; doi:10.3389/fonc.2025.1485006)
Supplement: Supplementary file 1 [file Table1.docx]

**Supplementary Table 1** the primer sequences.

| **Sequence** | **Name** |
| --- | --- |
| AAAGGGTCATCATCTCTG | GAPDH-F |
| GCTGTTGTCATACTTCTC | GAPDH-R |
| GCTACAAACAGCCAAGAAG | CHMP4A-F |
| GTGGATAATGTCCCGTCAG | CHMP4A-R |
| TGCTTCCTCCGTGTCTGTC  GCATTGTGAACCGCATCTC | IFFO1-F  IFFO1-R |
| TGCCTGGTCTGGCTGATTC | ANKRD10-F |
| AGCGAGCTGCCTTGTGAAT | ANKRD10-R |
| TGTGGGACCTGTCGCTGTG | ZDHHC11-F |
| GCCGAGTGGCAACGGAAAA | ZDHHC11-R |
| GAGCCGTAACCGTATTGCC | CLPB-F |
| CCGTCCCAGAAACTCATCC | CLPB-R |
| CACGGAGCAAATCCTAACC | ANKMY1-F |
| CTTCACAACCAGCTCATTCC | ANKMY1-R |
| CTGGGCAGAATGGAAGGAT | TCAP-F |
| CTGCTGGTGGTAGGTCTCATG | TCAP-R |
| AAGTTTGCCATGAGTCCAT | POLG2-F |
| GTTCCACAGGGTTTCTATT | POLG2 -R |
